# Stimulus-dependent Maximum Entropy Models of Neural Population Codes

**DOI:** 10.1371/journal.pcbi.1002922

**Published:** 2013-03-14

**Authors:** Einat Granot-Atedgi, Gašper Tkačik, Ronen Segev, Elad Schneidman

**Affiliations:** 1 Department of Neurobiology, Weizmann Institute of Science, Rehovot, Israel; 2 Institute of Science and Technology Austria, Am Campus 1, Klosterneuburg, Austria; 3 Faculty of Natural Sciences, Department of Life Sciences and Zlotowski Center for Neuroscience, Ben Gurion University of the Negev, Be'er Sheva, Israel; Indiana University, United States of America

## Abstract

Neural populations encode information about their stimulus in a collective fashion, by joint activity patterns of spiking and silence. A full account of this mapping from stimulus to neural activity is given by the conditional probability distribution over neural codewords given the sensory input. For large populations, direct sampling of these distributions is impossible, and so we must rely on constructing appropriate models. We show here that in a population of 100 retinal ganglion cells in the salamander retina responding to temporal white-noise stimuli, dependencies between cells play an important encoding role. We introduce the stimulus-dependent maximum entropy (SDME) model—a minimal extension of the canonical linear-nonlinear model of a single neuron, to a pairwise-coupled neural population. We find that the SDME model gives a more accurate account of single cell responses and in particular significantly outperforms uncoupled models in reproducing the distributions of population codewords emitted in response to a stimulus. We show how the SDME model, in conjunction with static maximum entropy models of population vocabulary, can be used to estimate information-theoretic quantities like average surprise and information transmission in a neural population.

## Author Summary

In the sensory periphery, stimuli are represented by patterns of spikes and silences across a population of sensory neurons. Because the neurons form an interconnected network, the code cannot be understood by looking at single cells alone. Recent recordings in the retina have enabled us to study populations of a hundred or more neurons that carry the visual information into the brain, and thus build probabilistic models of the neural code. Here we present a minimal (maximum entropy) yet powerful extension of well-known linear/nonlinear models for independent neurons, to an interacting population. This model reproduces the behavior of single cells as well as the structure of correlations in neural spiking. Our model predicts much better the complete set of patterns of spiking and silence across a population of cells, allowing us to explore the properties of the stimulus-response mapping, and estimate the information transmission, in bits per second, that the population carries about the stimulus. Our results show that to understand the code, we need to shift our focus from reproducing single-cell properties (such as firing rates) towards understanding the total “vocabulary” of patterns emitted by the population, and that network correlations play a central role in shaping the code of large neural populations.

## Introduction

Neurons represent and transmit information using temporal sequences of short stereotyped bursts of electrical activity, or spikes [1]. Much of what we know about this encoding has been learned by studying the mapping between stimuli and responses at the level of single neurons, and building detailed models of what stimulus features drive a single neuron to spike [2]–[4]. In most of the nervous system, however, information is represented by joint activity patterns of spiking and silence over populations of cells. In a sensory context, these patterns can be thought of as codewords that convey information about external stimuli to the central nervous system. One of the challenges of neuroscience is to understand the neural *codebook*—a map from the stimuli to the neural codewords—a task made difficult by the fact that neurons respond to the stimulus neither deterministically nor independently.

The structure of correlations among the neurons determines the organization of the code, that is, how different stimuli are represented by the population activity [5]–[8]. These correlations also determine what the brain, having no access to the stimulus apart from the spikes coming from the sensory periphery, can learn about the outside world [9]–[11]. The source of these correlations, which arise either from the correlated external stimuli to the neurons, from “shared” local input from other neurons, or from “private” independent noise, has been heavily debated [12]–[15]. In many neural systems, the correlation between pairs of (even nearby or functionally similar) neurons was found to be weak [16]–[18]. Similarly, the redundancy between pairs in terms of the information they convey about their stimuli was also typically weak [19]–[21]. The low correlations and redundancies between pairs of neurons therefore led to the suggestion that neurons in larger populations might encode information independently [22], which was echoed by theoretical ideas of maximally efficient neural codes [23]–[25].

Recent studies of the neural code in large populations have, however, revealed that while the typical pairwise correlations may be weak, larger populations of neurons can nevertheless be strongly correlated as a whole [18], [26]–[33]. Maximum entropy models of neural populations have shown that such strong network correlations can be the result of collective effects of pairwise dependencies between cells, and, in some cases, of sparse high-order dependencies [18], [34]–[36]. Most of these studies have characterized the strength of network effects and spiking synchrony at the level of the total *vocabulary* of the population, i.e. the distribution of codewords averaged over all the stimuli. It is not immediately clear how these findings affect stimulus encoding, where one needs to distinguish the impact of correlated stimuli that the cells receive (“stimulus correlations”), from the impact of co-variance of the cells conditional on the stimulus (“noise correlations”). For small populations of neurons, it has been shown that taking into account correlations for decoding or reconstructing the stimulus can be beneficial compared to the case where correlations are neglected (e.g. [35], [37]–[40]). Similarly, generalized linear models highlighted the importance of dependencies between cells in accounting for correlations between pairs and triplets of retinal ganglion cell responses [41].

Here we present a new encoding model that allows us to study in fine detail the codebook of a large neural population. We define the *codewords* to be the joint activity patterns of the population in time windows whose duration reflects the typical width of the cross-correlation of spiking between pairs of neurons. Importantly, this model gives a joint probability distribution over the activity patterns of the whole population for a given stimulus, while capturing both the stimulus and noise correlations. This new model belongs to a class of maximum entropy models with strong links to statistical physics [27], [42]–[53] and is directly related to maximum entropy models of neural vocabulary [18], [27]–[32], allowing us to estimate the entropy and its derivative quantities for the neural code. In sum, the maximum entropy framework enables us to progress towards our goal of focusing attention on the level of joint patterns of activity, rather than capturing low-level statistics (e.g., the individual firing rates) of the neural code alone.

We start by showing that linear-nonlinear (LN) models of retinal ganglion cells responding to spatially unstructured stimuli capture a significant part of the single neuron response, but still miss much of the detail; in particular, we show that they fail to capture the correlation structure of firing among the cells. We next present our new *stimulus-dependent maximum entropy* (SDME) model, which is a hybrid between linear-nonlinear models for single cells and the pairwise maximum entropy models. Applied to groups of 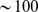 neurons recorded simultaneously, we find that SDME models outperform the LN models for the stimulus-response mapping of single cells and, crucially, give a significantly better account of the distribution of codewords in the neural population.

## Results

**Figure 1:**
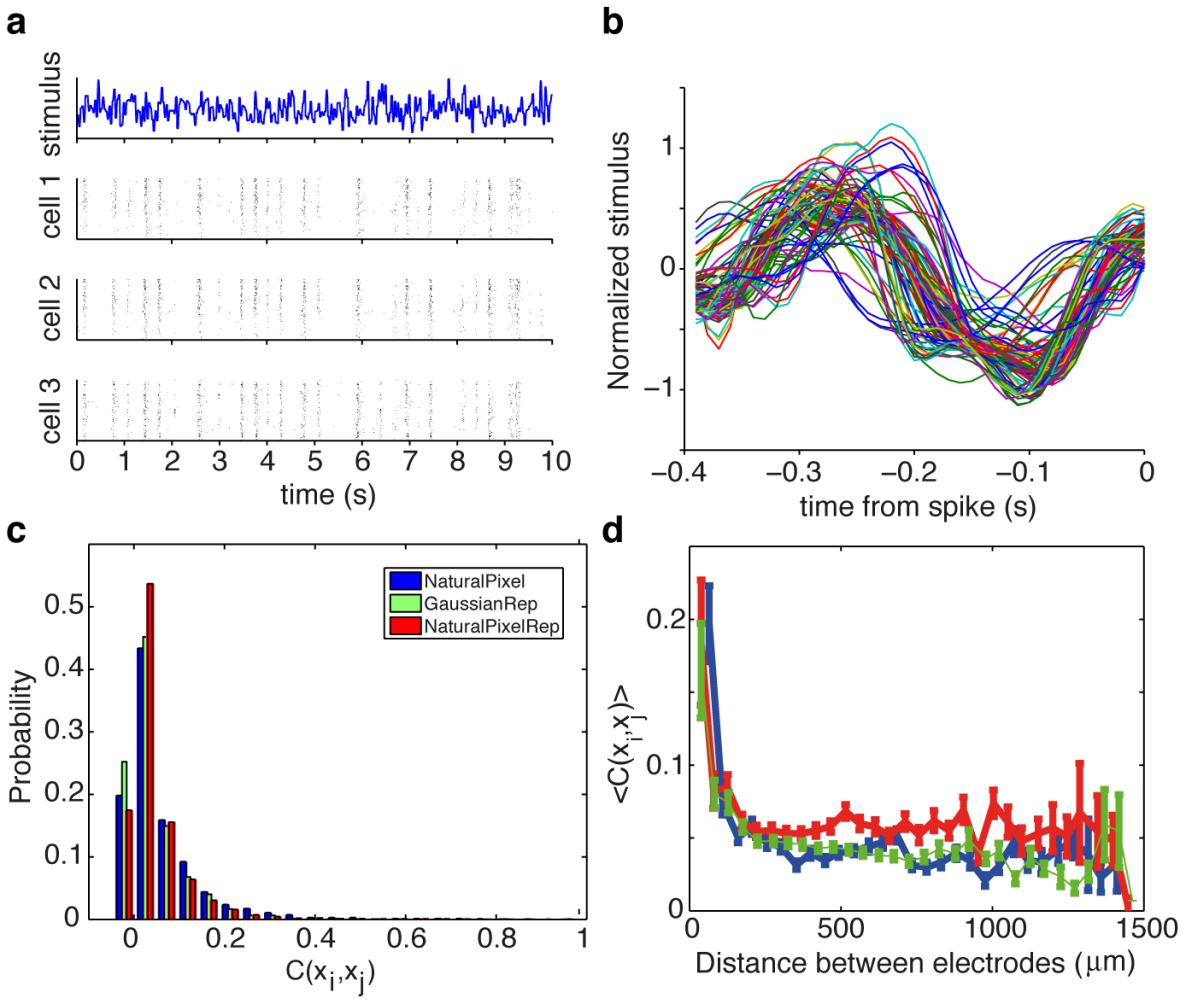
Response of a large population of ganglion cells to a 10 s long repeated visual stimulus. (**a**) White noise uncorrelated Gaussian stimulus presented at 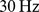 and the spiking patterns of 3 cells to repeated presentations of the stimulus. (**b**) Spike-trigerred averages of 110 simultaneously recorded cells; a subset of 100 cells was chosen for further analysis. (**c**) The histogram of pairwise correlation coefficients between cells for repeated Gaussian white noise stimulus (green). For comparison, the statistics of the response on repeated natural pixel movie (red), and non-repeated natural pixel movie (blue) is also shown, as documented in Ref. [35]. The significance cutoff for correlation coefficients is 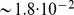, 95% of correlations are above this cut (see Methods). (**d**) Average pairwise correlation coefficient between cells as a function of the distance (mean and std are across pairs of cells at a given distance).

We recorded the simultaneous spiking activity of 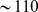 ganglion cells from the salamander retina [54], presented with repeats of a 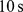 long full-field flicker (“Gaussian FFF”) movie, where the light intensity on the screen was sampled independently from a Gaussian distribution with a frequency of 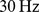 (Fig. 1a). This “frozen noise” stimulus was repeated 726 times, for a total of 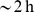 of stimulation. Most of the recorded cells exhibited temporal OFF-like behaviors (Fig. 1b). We chose for further analysis 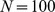 cells that were reliably sorted, demonstrated a robust and stable response over repeats, and generated at least 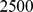 spikes during the course of the experiment. We also left out the first 100 repeats of the stimulus, when the retina was still adapting, to ensure stationarity (see Methods). To construct the population response codewords, we discretized time into 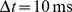 bins, and represented the activity of the neurons in response to the stimulus as binary patterns in each of the time bins. If neuron 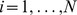 was active in time bin 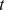, we denoted a spike (or more spikes) as 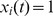, and 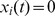 if it was silent. In this representation, the whole experiment yielded a total of about 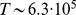 100-bit samples. Maximum entropy models are defined by a choice of constrained statistics over the ensemble of codewords and stimuli, as we discuss below; our ability to estimate these reliably from data is thus a key systematic issue, which we address in the Methods section.

All models of the population responses were fitted based on one half of our data (313 training repeats), and evaluated (tested) on the other half of repeats; overall, the train and test data were each almost 1 hr long. While fitting the stimulus-dependent maximum entropy model can be done using non-repeated stimuli, assessing the performance of the models requires many repeated presentations of the same stimulus to quantify both single cell and in particular population spiking patterns, as well as noise entropy and mutual information. Unlike for single neurons (which are fully characterized by their firing rate), in the case of large populations, capturing well the very high-dimensional distribution of codewords given the stimulus, 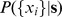, is a non-trivial problem, as we show below. Because we were interested in models of codeword distributions, we chose the experimental design that maximizes the number of repeats rather than the duration of the stimulus; consequently, we examined how the models generalize across stimulus repeats rather than across different stimuli. Despite the limited duration of the stimulus segment, the large number of repeats nevertheless enabled us to recover smooth estimates of the linear filters (Fig. 1b). Furthermore, because of the way we construct our maximum entropy models, these linear filters are *the same* for all the models considered, so the performance of the models cannot differ due to the differences in modeled stimulus sensitivities. With this setup, we are therefore able to fairly compare the performance and generalization of various models of joint population activity given the stimulus.

### Conditionally independent Linear-Nonlinear models for a neural population

**Figure 2:**
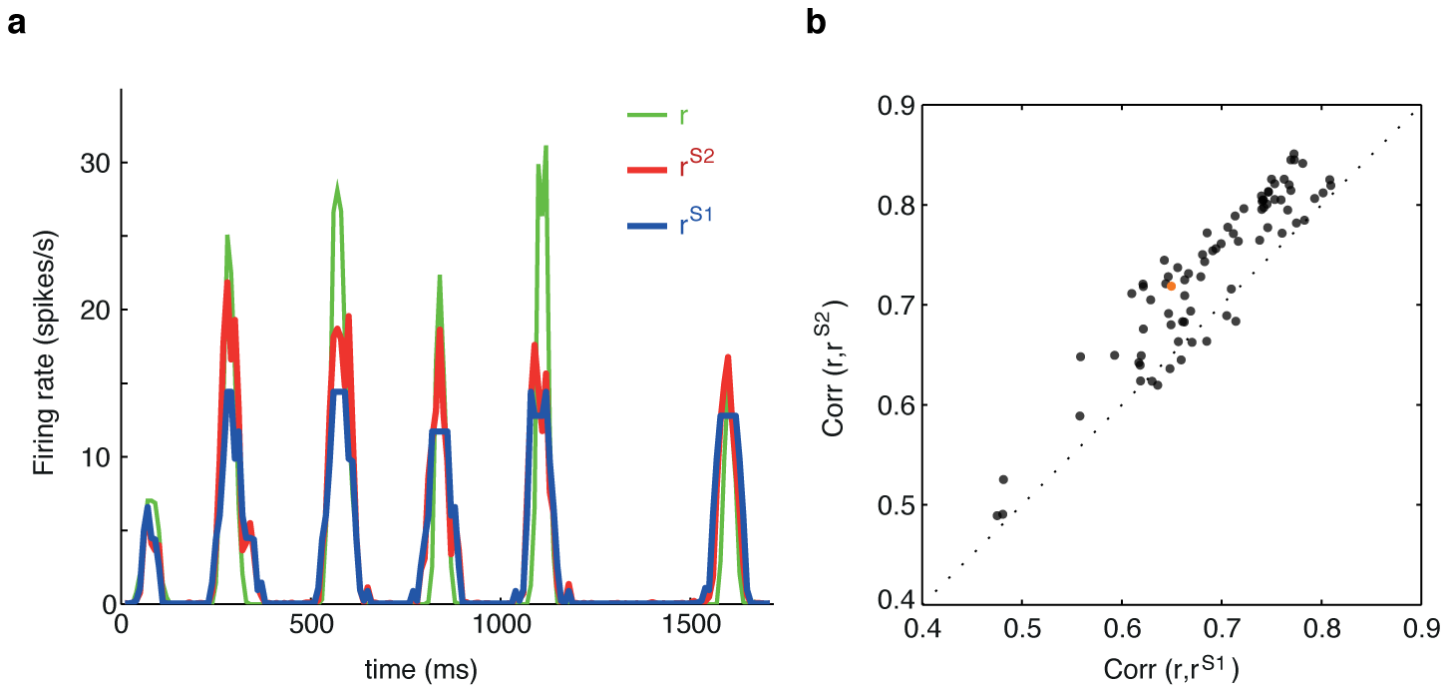
Pairwise SDME (S2) model predicts the firing rate of single cells better than conditionally independent LN (S1) models. (**a**) Example of the PSTH segment for one cell (green), the best prediction of the S1 model (blue) and of the S2 model (red). (**b**) Correlation coefficient between the true PSTH and S2 model prediction (vertical axis) vs. the correlation between the true PSTH and the S1 model prediction (horizontal axis); each plot symbol is a separate cell, dotted line shows equality. S2 significantly outperforms S1 (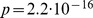, paired two-sided Wilcoxon test). The neuron chosen in panel (a) is shown in orange.

Using repeated presentations of the same movie, we estimated the average response of each of the cells across repeats, 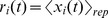, or the peri-stimulus time histogram (PSTH). Following Refs. [4], [55], we fitted a linear-nonlinear model for each of the cells in the experiment, so that the resulting model for the population as a whole is a set of uncoupled, conditionally independent LN neurons that we denote together as a ‘S1’ model (the reason for this notation will be explained later). The predicted rate of every neuron is then 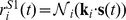, where 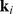 is a linear filter matched for the 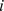-th cell, 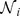 is its point-wise nonlinear function, and 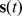 is the stimulus fragment from time 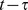 until 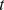 (here we used 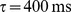, making 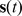 a vector of light intensities with 40 components). Linear filters were reconstructed using reverse correlation (spike-triggered average), and nonlinearities were obtained by histograming 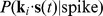 into 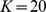 adaptively-sized bins and obtaining 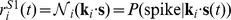 by inverting 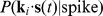 using Bayes' rule. These LN models captured most of structure of the PSTH, yet as the example cell in Fig. 2a shows, they often misestimated the exact firing rates of the neuron, or sometimes even missed parts of the neural response altogether. For the Gaussian FFF, the normalized (Pearson) correlation between the measured and predicted PSTH, 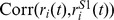, was 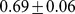 (mean 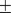 std across 100 cells).

The performance gap of the canonical LN models in predicting single neuron responses suggests that either the single-neuron models need to be improved to account for the observed behavior, or that interactions between neurons play an important encoding role and need to be included. Clearly, the firing rate prediction performance can be improved for single neurons by models with higher-dimensional stimulus sensitivity (e.g. [55], [56]) or dynamical aspects of spiking behavior (e.g. [57], [58]). However, previous work (and results below) demonstrated that even conditionally-independent models which by construction perfectly reproduce the firing rate behavior of single cells, often fail to capture the measured correlation structure of firing between pairs of cells, as well as higher-order statistical structure [18].

We therefore sought a model of the neural code that would be able to reproduce the correlation structure of population codes. We asked whether a model that combined the LN (receptive-field based) aspect of single cells with the interactions between cells, could give a better account of the neural stimulus-response mapping. Importantly, the new model should capture not only the firing rate of single cells but also accurately predict the full distribution of the joint activity patterns across the whole population. Because the joint distributions of activity are high-dimensional (e.g., the distribution over codewords across the duration of the experiment, 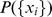, has 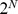 components), this is a very demanding benchmark for any model.

### A Stimulus Dependent Maximum Entropy model for a neural population

We propose the simplest extension to the conditionally-independent set of LN models for each cell in the recorded population, by including pairwise couplings between cells, so that the spiking of cell 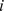 can increase or decrease the probability of spiking for cell 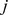 [59], [60]. Importantly, in contrast to previous models, we introduce this coupling so that the resulting model is a maximum-entropy model for 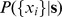, the conditional distribution over population activity patterns given the stimulus. We recall that the maximum entropy models give the most parsimonious probabilistic description of the joint activity patterns, which perfectly reproduces a chosen set of measured statistics over these patterns, without making any additional assumptions [61].

Specifically, we construct a model that relies only on the measured overall correlations between pairs of neurons, which can be reliably estimated from experimental data (see Methods). We find that (i) the pairwise correlations between cells in response to the Gaussian FFF movie are typically weak but significantly different from zero (Fig. 1c, consistent with previous reports [18], [27], [32]); (ii) the correlation in neural activities shows a fast decay with distance despite the infinite correlation length of the stimulus, but the decay does not reach zero correlation even at relatively large distances (Fig. 1d). This salient structure, along with any other potential statistical correlation at the pairwise order, is characterized by the covariance matrix of activities, 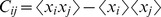, where the averages are taken across time and repeats.

We start by introducing the least structured (maximum entropy) distribution of the population responses to stimuli, by treating each time point along the stimulus separately; since every moment of time maps uniquely into one stimulus, we start by building the model of the response given time. We thus find 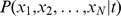 that reproduces exactly the observed average firing rate for each time bin 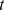 in the stimulus and for each neuron 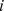, 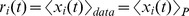, as well as the overall covariance matrix 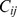 between all pairs of cells (c.f. [62]). Thus, we seek 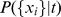 that maximizes 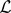:
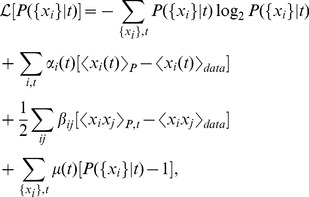
(1)where the subscript to brackets 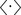 denotes whether the averaging is done over the maximum entropy distribution (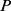), or over the recorded data; Lagrange multipliers 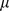 ensure that the distributions are normalized. This is an optimization problem for parameters 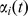 and 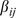, which has a unique solution since the entropy is convex. The functional form of the solution to this optimization problem is well-known and in our case it can be written as
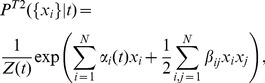
(2)where the individual time-dependent parameters for each of the cells, 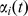, and the stimulus-independent pairwise interaction terms 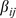, are set to match the measured firing rates 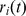 and the pairwise correlations 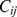; 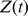 is a normalization factor or partition function for each time bin 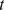, given by 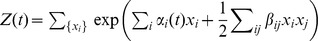.

The *pairwise time-dependent maximum entropy (pairwise TDME or T2) model* in Eq. (2) is equivalent to an Ising model from physics, where the single-cell parameters are time-dependent local fields acting on each of the neurons (spins), and static (stimulus-independent) infinite-range interaction terms couple each pair of spins. In the limit where interactions go to zero, 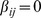, the model in Eq. (2) becomes the full conditionally-independent model, itself a *first-order time-dependent maximum entropy model* that reproduces exactly the firing rate of every neuron, 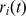:
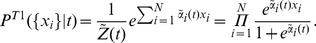
(3)In this case the probability distribution factorizes, and the solution for 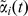 and 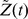 becomes trivially computable from the firing rates, 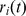. For time bins 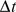 that are short enough to contain 0 or 1 spike (as we have assumed throughout), 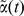 is given by 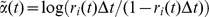. Consistent with our previous notation, we denote this full conditionally-independent model as **T1**.

Time-dependent maximum entropy models are powerful, since they make no assumption about how the stimulus drives the response; they often serve as useful benchmarks for other models (especially the T1 model). On the other hand, these models require repeated stimulus presentations to fit, involve a number of parameters that grows linearly with the duration of the stimulus, do not generalize to new stimuli, and do not provide an explicit map from the stimuli to the responses.

We therefore present a more particular form of the model of Eq. (2) that, **(i)**, would give an explicit description of stimulus-dependent distribution of population patterns; **(ii)**, would generalize to new stimuli; **(iii)**, could be directly compared to the uncoupled LN models; and **(iv)**, would not require repeats of the same stimulus to fit. Specifically, rather than having an arbitrary time-dependent parameter for every neuron for each time bin, 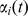, we assume that this dependence takes place through the stimulus projection alone, i.e. 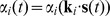. This is analogous to an LN model, where the neural firing depends on the value of the stimulus projection onto the linear filter 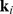. This choice is made for simplicity; this model can be generalized to, e.g., neurons that depend on two linear projections of the stimulus, by making 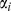 depend jointly on 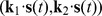, although such models would be progressively more difficult to infer from data.

Concretely, we estimated the linear filter 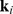 for each cell 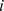 using reverse correlation, and convolved the filter with the stimulus sequence, 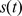, to get the “generator signal” 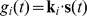. We then looked for the maximum entropy probability distribution 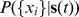, by requiring that the average firing rate of every cell given the generator signal is the same in the data and under the model, i.e. 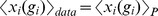 (see Methods); as before, we also required the model to reproduce the overall covariance between all pairs of cells, 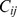. This yields a *pairwise stimulus-dependent maximum entropy (pairwise SDME or S2) model*, which takes the following form:
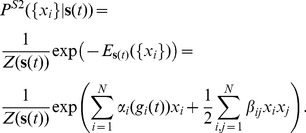
(4)The parameters of this model are: 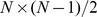 couplings 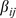, 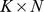 parameters 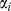, and a linear filter 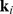 for each cell; these parameters define the energy function 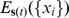 of the model. We used a Monte Carlo based gradient descent learning procedure to find the model parameters 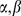 numerically (see Methods; note that the problem is still convex with a single solution for the parameter values).

By construction, the S2 model exactly reproduces the covariance of activities, 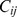, between all pairs of cells, and also the LN model properties of every cell: an arbitrary nonlinear function 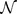 can be encoded by properly choosing how parameters 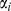 depend on the linear projections of the stimulus, 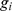. We can construct a maximum entropy model with 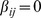 (no constraints on the pairwise correlations 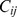). The result is a set of uncoupled (conditionally independent) LN models:
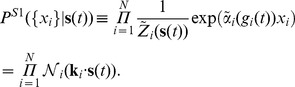
(5)


**Figure 3:**
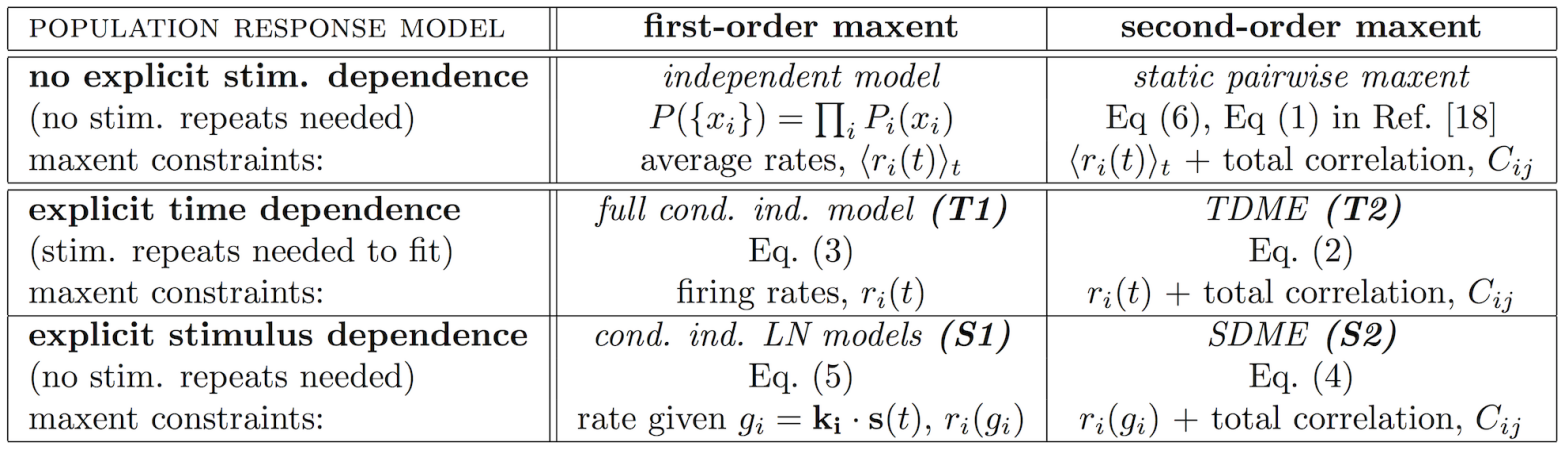
An overview of maximum entropy encoding models. The explicit dependence of single-neuron terms (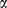, vertical axis, ‘T’ or ‘S’), and the absence or presence of pairwise terms (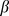, horizontal axis, ‘1’ or ‘2’), together define the type of the maximum entropy model (e.g. pairwise SDME is ‘S2’). For completeness, the first row of the table includes static maximum entropy models of population vocabulary, 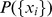, which have no explicit stimulus dependence. Full conditionally independent model (T1) reproduces exactly the instantaneous firing rate of every neuron, and thus fully captures the stimulus sensitivity, history effects, and adaptation on a single neuron level; for experimentally recorded rasters with stimulus repeats, simulated T1 rasters are often generated by taking the original data and, at each time point and for every neuron, randomly permuting the responses recorded on different stimulus repeats. “Total correlation” is the pairwise correlation matrix of activities, 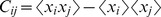, averaged over all repetitions and all times in the experiment.

Fig. 3 shows all the models in a systematic way: the pairwise time-dependent maximum entropy (T2) model of Eq. (2) is an extension of conditionally independent (T1) model that additionally reproduces the measured pairwise correlations between cells. In a directly analogous way, the pairwise stimulus-dependent maximum entropy (S2) model of Eq. (4) is an extension to the set of uncoupled LN models (S1), Eq. (5), that additionally reproduces the measured pairwise correlations between cells. Because 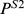 (Eq. 4) agrees with 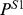 (Eq. 5) exactly in all constrained single-neuron statistics, any improvement in prediction of the S2 model, be it in the firing rate or the codeword distributions, can be directly ascribed to the effect of the interaction terms, 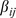.

An alternative approach to describing the joint response of large populations of neurons to external stimuli has been presented in Ref. [41]. The Generalized Linear Model (GLM) gives a generative model from which one can sample simulated responses to new stimuli, relying on activity history and temporal dependencies between cells, but assuming conditional independence within any given time bin. We compare the advantages of the two approaches in the Discussion below, but briefly emphasize here that a key difference is that GLM does not present an explicit probability distribution over codewords (that are defined for temporal bins significantly longer than those of the GLMs), which is central for the analysis of the neural code we present below.

### Pairwise SDME (S2) model outperforms conditionally independent models in describing single cell responses and joint patterns of activity

To assess the accuracy of different stimulus-dependent models, and, in particular, of the contribution of the interactions between cells, we fitted and quantified the performance of the uncoupled LN models (S1) and the pairwise SDME model (S2). At the level of single neurons, we found that the S2 model predicted the firing rates better than the S1 model (see e.g. Fig. 2a), with the normalized correlation coefficient between the true and predicted firing rate, 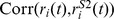 reaching 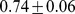 (mean 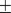 std across 100 cells), as shown in Fig. 2b.

**Figure 4:**
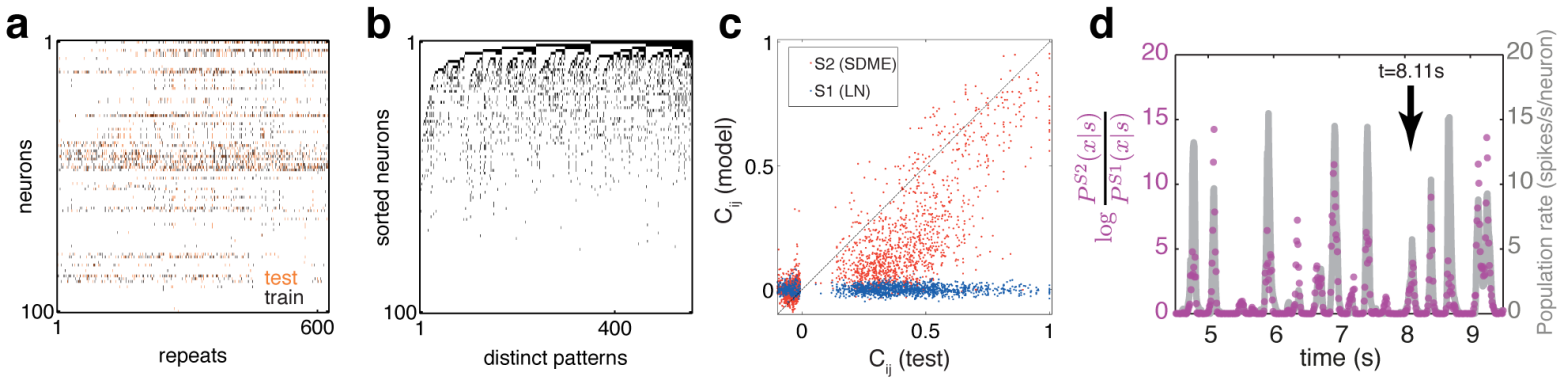
Pairwise SDME (S2) model predicts population activity patterns for 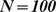 neurons better than conditionally independent LN (S1) models. (**a**) The activity raster for 100 neurons across 626 repeats of the stimulus at a point in time where the retina is moderately active (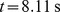). Dots represent individual spikes; training repeats denoted in black, test repeats in orange. (**b**) The diversity in retinal responses in a. Shown are all distinct patterns; their number is comparable to the number of repeats. Neurons are resorted by their instantaneous firing rate (high rate = top, low rate = bottom). (**c**) S2 model fit on the training repeats predicts the reliably estimated correlation coefficients between pairs of neurons at various time points where the retina is active. We identify all correlation coefficients whose value can be estimated from data with less than 25% relative error across many splits of the repeats into two halves. The value of these correlation coefficients is estimated on the test set (horizontal axis) and compared to the model prediction (vertical axis). (**d**) The log-likelihood ratio of the population firing patterns under the S2 model and under the S1 model, shown as a function of time (violet dots, scale at left) for an example (test) stimulus repeat. For reference, the average population firing rate is shown in grey (scale at right). The arrow denotes the time bin displayed in a, b.

The differences between the S2 and the S1 models become more striking at the level of the activity patterns of the whole population. Figs. 4a,b show the complex structure of the population activity patterns across all 626 repeats at a particular moment in time. During times when the population is active, it generates a wide diversity of patterns in response to the same stimulus; even with hundreds of repeats, these distributions cannot be empirically sampled. Nevertheless, the large number of repeats suffices to identify and estimate reliable low-order marginals of these distributions, in particular, the correlations between the pairs of neurons at various points in time. The wide range of magnitudes of these reliably estimated correlations shows that a number of neuronal pairs are far from conditionally independent. As shown in Fig. 4c, the S2 model captures a significant fraction of this correlation structure on a timebin-by-timebin basis (on test data); clearly, the S1 model fails at this task.

**Figure 5:**
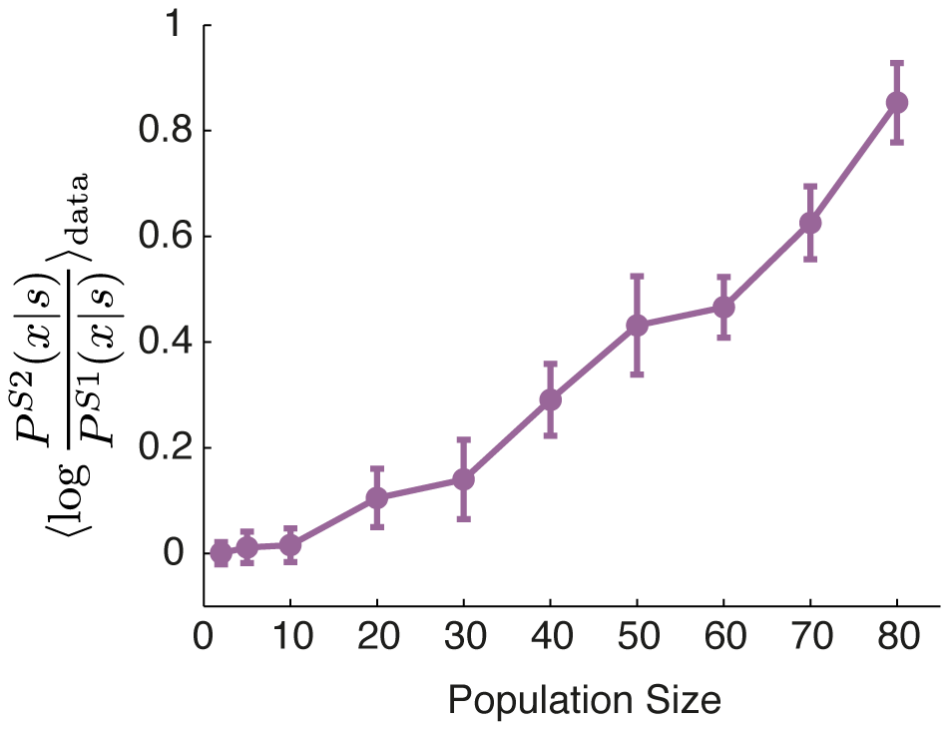
The performance of the SDME (S2) model relative to conditionally independent LN (S1) models. The average log likelihood ratio between the S2 and the S1 models evaluated on the test set, as a function of the population size, 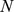 (error bars = std over 10 randomly chosen groups of neurons at that 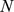).

We found that S2 is orders of magnitude better in predicting the population neural responses to stimuli. This is quantified in Fig. 4d, which compares S1 and S2 through the log-likelihood ratio, 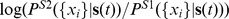, for the population activity patterns 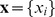 under the two models. These differences are large in particular for those stimuli that elicit a strong response, that is, precisely where the response consists of synchronous spiking and the structure of the codewords can be nontrivial. Fig. 5 summarizes these results by showing the average log-likelihood ratio over all testing repeats, emphasizing that the difference between the models becomes particularly apparent for groups of more than 20 cells.

**Figure 6:**
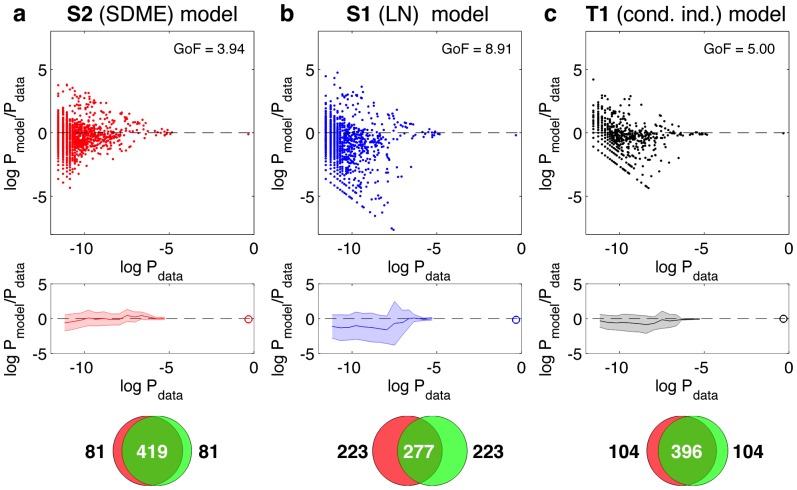
The performance of various models in accounting for the total vocabulary of the population, 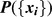
 **.** The results for the S2 model are shown in (**a**), the results for the S1 model in (**b**), and the results for a full conditionally independent model (T1) in (**c**). The first row displays the log ratio of model to empirical probabilities for various codewords (dots), as a function of that codeword's empirical frequency in the recorded data. The model probabilities were estimated by generating Monte Carlo samples drawn from the corresponding model distributions; only patterns that were generated in the MC run as well as found in the recorded data are shown. GoF quantifies the deviation between true and predicted 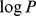 of the non-silent codewords shown in the plot; smaller values indicate better agreement (see Methods). The second row summarizes this scatterplot by binning codewords according to their frequency, and showing the average log probability ratio in the bin (solid line), as well as the 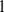 std scatter across the codewords in the bin (shaded area). The highly probable all-silent state, 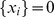, is shown separately as a circle. The third row shows the overlap between 500 most frequent patterns in the data and 500 most likely patterns generated by the model (see text). Models were fit on training repeats; comparisons are done only with test repeats data.

We next examined how well various models of the neural codebook, 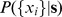, explain the total vocabulary, that is, the distribution of neural codewords observed across the whole duration of the experiment, 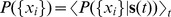. Despite the nominally large space of possible codewords—much larger than the total number of samples in the experiment (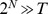)—the sparsity of spikes and the correlations between neurons restrict the vocabulary to a much smaller set of patterns. Some of these occur many times during our stimulus presentation, allowing us to estimate their empirical probability, 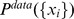, directly from the experiment, and compare it to the model prediction [35]. The most prominent example of such frequently observed codewords is the silent pattern, 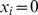, which is seen 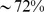 of the time. Fig. 6 shows the likelihood ratio of the model probability and empirical probability for various codewords observed in the test part of the experiment, as a function of the rate at which these codewords appear. Here we used an additional model for comparison, i.e., the full conditionally-independent model (T1), where every cell is described in terms of time-dependent firing rate. The S2 model in Fig. 6a strongly outperforms the S1 model in Fig. 6b, and has a slightly better performance than the T1 model (Fig. 6c), despite the fact that the latter is determined by 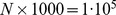 parameters, the firing rates of every cell in every time bin. Quantitatively, the per-codeword log-likelihood of the test data under S1 model is 5.30, under T1 model 4.34, under S2 model 4.12, under empirically sampled distribution on the training set 4.02, while the lower bound on the log-likelihood (obtained when the “model” are the true empirical frequencies on the test set) is 2.98 (see Methods).

On average, S2 predicts the probabilities of the patterns of activity with minimal bias, and with a standard deviation of 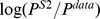 of about 1; the S1 model in comparison is biased and has a spread that is more than twice as large. Even more striking is the fact that S1 assigns very low probabilities to some codewords such that they were never generated during our Monte Carlo sampling (and are therefore not even shown in scatterplots of Fig. 6), although they were frequently observed in the experiment. This discrepancy is quantified by enumerating the 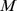 most probable patterns in the data and in the model (by sampling, see Methods), and measuring the size of the intersection of the two sets of patterns. In other words, we ask if the model is even able to access all the patterns that one is likely to record in the experiment. As shown in the bottom of Fig. 6, S2 does well on this task, with 419 codewords in the intersection of the 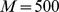 most likely patterns in the data and the model. This is a much better performance than the S1 model, and a little better than for the T1 model (which has many more parameters). We emphasize that all these comparisons were done on test data only, so that the models had to generalize over the large diversity of patterns where some of the patterns seen in the training set might never occur on the testing set and vice versa (see Fig. 4a,b).

**Figure 7:**
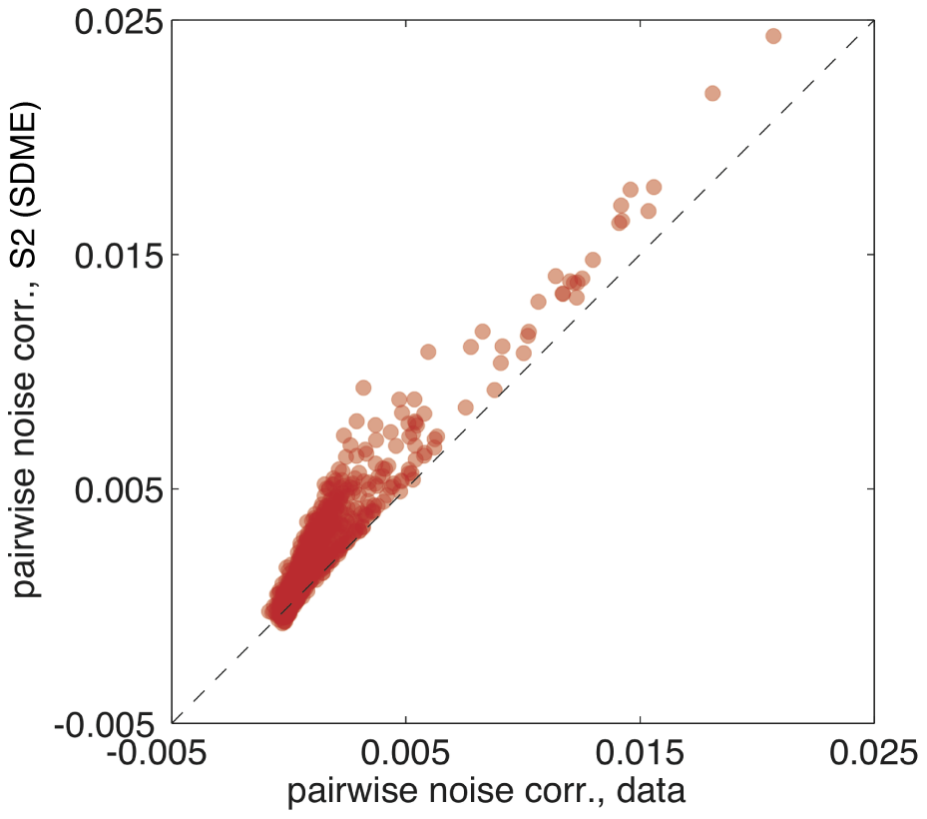
Measured vs predicted noise correlations for the pairwise SDME (S2) model. Noise correlation (see text) is estimated from recorded data for every pair of neurons, and plotted against the noise correlation predicted by the S2 model (each pair of neurons = one dot; shown are 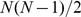 dots for 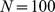 neurons; for significantly correlated pairs, the slope of the best fit line is 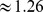, with 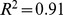). Conditionally independent models predict zero noise correlation for all pairs.

The S2 model was constructed to capture exactly the total pairwise correlation in neuronal spiking, 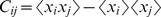. With repeated stimulus, this total correlation can be broken down into the signal and noise components. The signal correlations, 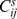, are inferred by applying the same formula as for the total correlation, but on the spiking raster where the repeated trial indices have been randomly and independently permuted for each time bin. This removes any correlation due to interactions between spikes on simultaneously recorded trials, and only leaves the correlations induced by the response being locked to the stimulus. The noise correlation, 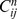, is then defined as the difference between the total and the signal components, 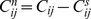. We calculated the noise correlations between all pairs in our 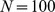 neuron dataset. By their definition, the conditionally independent models cannot reproduce 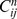, which are always zero for those models. To assess the performance of the S2 model, we drew samples from our model distribution using a Monte Carlo simulation and compared the noise correlations in the simulated rasters to the true noise correlations. The model prediction is tightly correlated with the measured values, as shown in Fig. 7. We observe a systematic deviation of 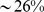, most likely because the assumed dependence on the stimulus through one linear filter per neuron is insufficient to capture the complete dependence on stimulus, thereby underestimating the full structure of stimulus correlation and inducing an excess in the noise correlation. Despite this, the degree of correspondence in noise correlations observed in Fig. 7 is telling us that the S2 model has clearly captured a large amount of noise covariance structure in neural firing at the network level.

### Interpretation of the functional interactions between cells in the pairwise SDME (S2) model

**Figure 8:**
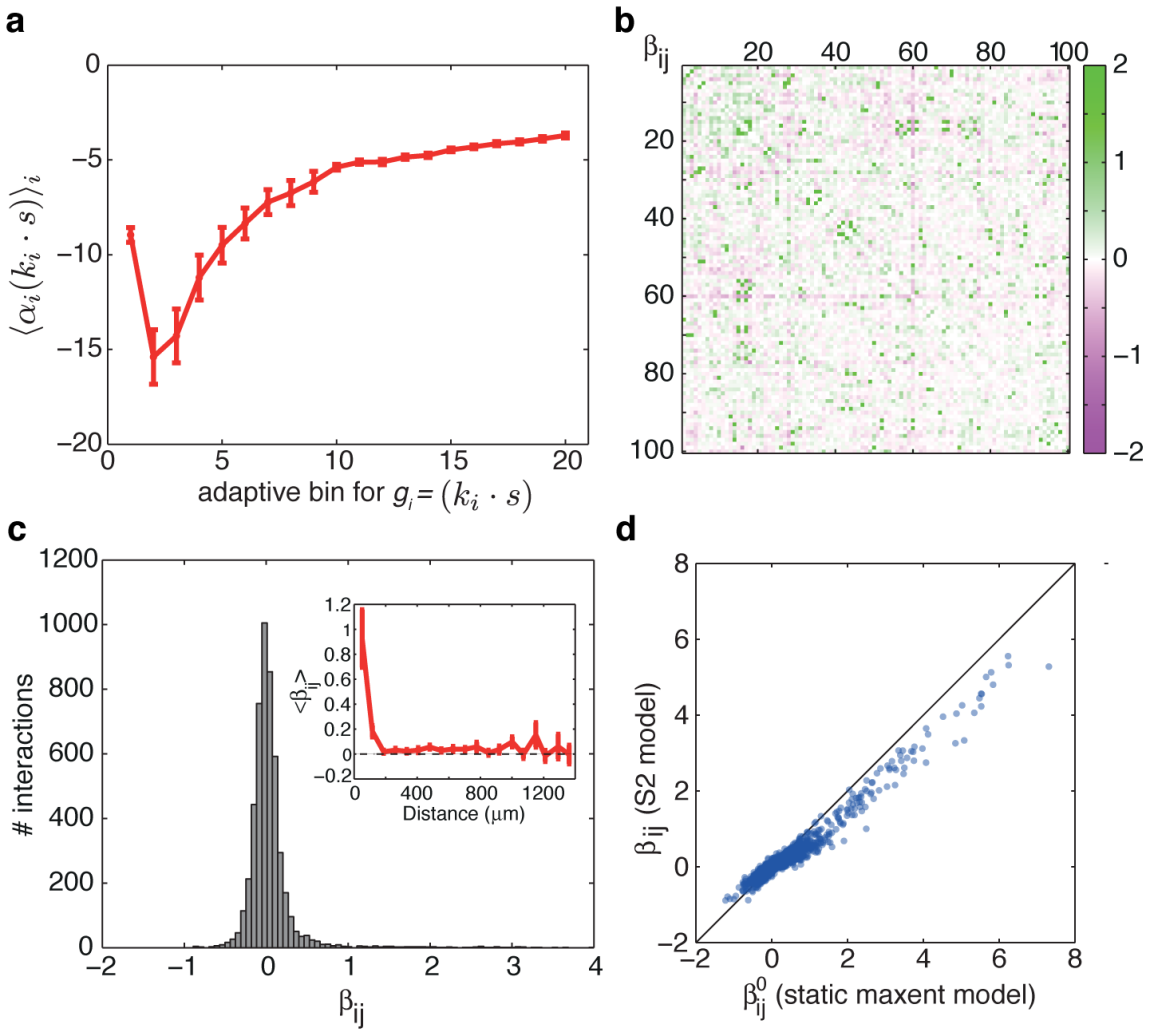
Pairwise SDME (S2) model parameters. (**a**) Average values of the LN-like driving term, 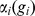, where 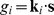, across all cells 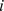 (error bars = std across cells), for each of the 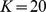 adaptive bins for 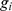 (see Methods). (**b**) Pairwise interaction map 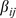 of the S2 model, between all 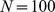 neurons in the experiment. (**c**) Histogram of pairwise interaction values from (b), and their average value as a function of the distance between cells (inset). (**d**) For each pair of cells 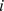 and 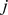, we plot the value of 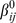 under the static maximum entropy model of Eq. (6) vs. the 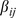 from the S2 model of Eq. (4).

How should we interpret the inferred parameters of the S2 model? LN models have a clear mechanistic interpretation in terms of the cell's receptive field and the nonlinear spiking mechanism. Here, similarly, the stimulus dependent part of the model for each cell, 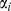, is a nonlinear function of a filtered version of the stimulus 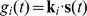; in the absence of neuron-to-neuron couplings, the nonlinearity of every neuron would correspond to 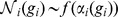, where 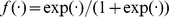, according to Eq. (5). The dependence of 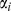 on the stimulus projection 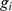 is similar across the recorded cells as shown in Fig. 8a; as expected, higher overlaps with the linear filter induce higher probability of spiking.

The pairwise interaction terms in the S2 model, 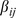, are symmetric, static, and stimulus independent by construction. As such, they represent only functional and not physical (i.e. synaptic) connections between the cells. Fig. 8b shows the pairwise interaction map for 100 cells; the histogram of their values (in Fig. 8c) reflects that they can be of both signs, but the distribution has a stronger positive tail, i.e. a number of cell pairs tend to spike together or be silent together with a probability that is higher than expected from their respective LN models. We can compare these interactions to the interactions of a static (non-stimulus-dependent) pairwise maximum entropy model for the population vocabulary [18], [28]:
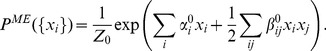
(6)In this model for the total distribution of codewords, there is no stimulus dependence, and the parameters 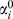 and 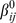 are chosen so that the distribution is as random as possible, while reproducing exactly the measured mean firing rate of every neuron 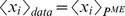, and every pairwise correlation, 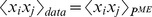, across the whole duration of the experiment.

Interestingly, we find that the pairwise interaction terms in the S2 model of Eq. (4) are closely related to the interactions in the static pairwise maximum entropy model of Eq. (6): S2 interactions, 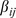, tend to be smaller in magnitude, but have an equal sign and relative ordering, as the static ME interactions, 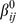. Some degree of correspondence is expected: an interaction between neurons 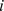 and 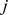 in the static ME model captures the combined effect of the stimulus and noise correlations, while in the corresponding S2 interaction, (most of) the stimulus correlation has been factored out into the correlated dynamics of the inputs to the neurons 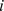 and 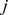, i.e. 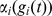 and 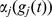. The surprisingly high degree of correspondence, however, indicates that even the interactions learned from static maximum entropy models can account for, up to a scaling factor, the pairwise neuron dependencies that are *not* due to the correlated stimulus inputs.

### Pairwise SDME (S2) model partitions the space of activity patterns into clusters that generalize to testing data

Figs. 4a,b show the richness of activity patterns produced in response to repeats of the same stimulus. While these patterns must encode the same information, it is not clear how this could be established by looking at the patterns alone (without prior knowledge that they were generated in response to the same stimulus), because of the high dimensionality of the pattern space. Is there a way to simplify this response space? We suggest one such approach here, motivated by the analogy to Ising models in statistical physics and the related similarities with the Hopfield networks [27], [32], [62], [63].

**Figure 9:**
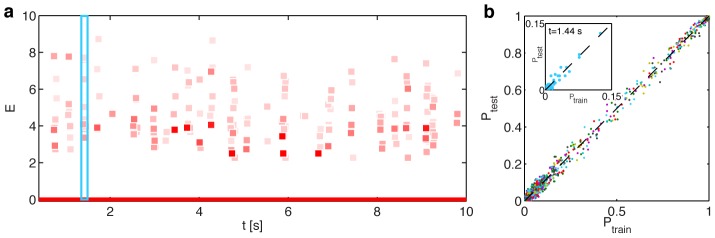
Clustering of response patterns into basins of attraction centered on meta-stable patterns generalizes across repeats. **a**) Every response pattern 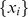 from data is assigned to its corresponding meta-stable pattern 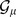 by descending on the energy landscape 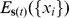 defined by the S2 model of Eq (4) until the local minimum is reached (see text). Across all test repeats and at each point in time (horizontal axis), we find the metastable states that are visited more than 30 times, plot their energy 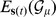 (vertical axis), and the number of repeats on which that metastable state is visited (shade of red). **b**) Inset: for 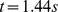 (blue rectangle in a), we plot the frequency of visit to each metastable state (dots) in the training set (horizontal) against the frequency in the test set (vertical). Main panel: the same analysis across all time bins (different colors) superposed, dashed line is equality.

At every instant in time, the probability of any activity pattern 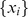 in the S2 model is fully specified by the distribution with an exponential form given by Eq. (4). In analogy to statistical physics, the exponent is the (negative) energy of the state 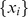. This energy function defines an instantaneous “energy landscape” over the space of all possible activity patterns. Minima in this landscape can be viewed as metastable patterns or attractors, and all activity patterns can be assigned to their respective attractors by descending on the energy landscape until the closest local minimum is reached, much like in the Hopfield network. In this way, the space of 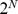 patterns is partitioned, at each point in time, into a number of domains centered on the metastable states. How useful is this representation of the response space? Using the S2 model fit on training repeats, we examined neural responses in every time bin across all testing repeats. We assigned each response pattern from testing data to its corresponding metastable state. Fig. 9a shows, as a function of time, all identified metastable states, their energies (i.e. the negative log probability of that state), and the number of repeats on which a pattern belonging to that state was emitted. This analysis still paints a rich, but already much simplified picture of the retinal responses, where many patterns are grouped into a small number of clusters centered on the metastable states. Interestingly, these assignments generalize very well: in Fig. 9b we independently identify the metastable states on testing and training sets for each time bin, assign all patterns seen in the experiment to these states, and count and compare how many times each state appears on testing and training repeats. Virtually all (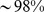) metastable states appearing in training repeats are found on testing repeats and vice versa, and this intersection is vastly larger than the intersection of the activity patterns themselves, a lot of which can appear only once in all 626 repeats. Moreover, the frequency with which patterns belonging to a particular metastable state occur is reproducible between the training and test data, suggesting that the partitioning of the high-dimensional activity space into clusters defined by the energy function of the S2 model is a productive dimensionality reduction method in this context.

### Pairwise SDME (S2) model reveals the strongly correlated nature of information encoding by large neural populations

The S2 model is an approximation to the neural codebook, 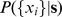, while the static ME model describes the population vocabulary, 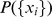. With these two distributions in hand, we can explore how the population jointly encodes the information about the stimulus into neural codewords—the joint activity patterns of spiking and silence. We make use of the fact that we can estimate the entropy of the maximum entropy distributions using a procedure of heat capacity integration, as explained in Refs. [27], [32] (see Methods). The information (in bits) that the codewords carry about the stimulus is then
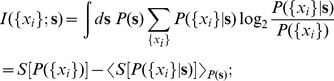
(7)that is, the information can be written as a difference of the entropy of the neural vocabulary, and the noise entropy (the average of the entropy of the codebook), where the entropy is 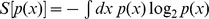. Because of the maximum entropy property of our model for 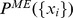, the entropy of our static pairwise model in Eq. (6) is an upper bound on the transmitted information; expressed as an entropy rate, this amounts to 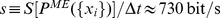.

**Figure 10:**
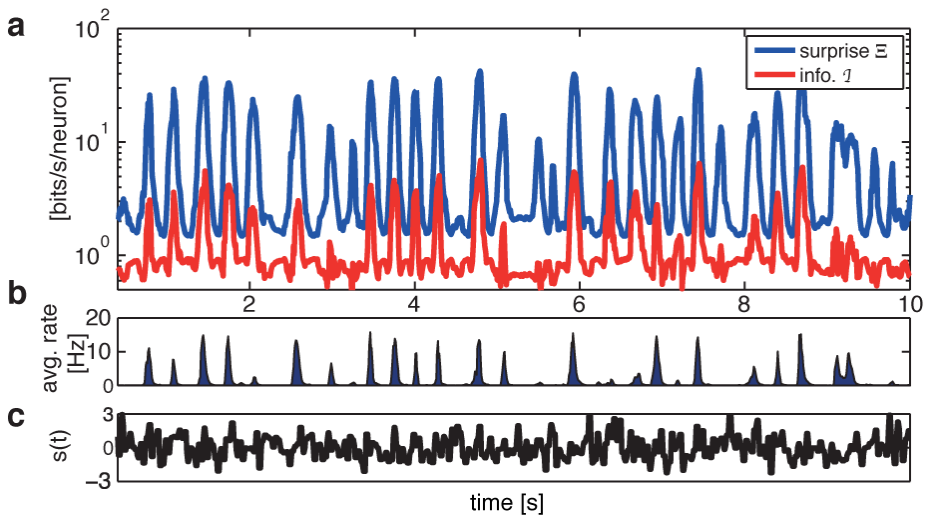
Surprise and information transmission estimated from the pairwise SDME (S2) model. (**a**) Surprise rate (blue) is estimated from the static ME and S2 models assuming independence of codewords across time bins. The instantaneous information rate (red) is the difference between the surprise and the noise entropy rate, estimated from the S2 model (see text). The information transmission rate is the average of the instantaneous information across time. (**b**) Population firing rate as a function of time shows that bursts of spiking strongly correlate with the bursts of surprise and information transmission in the population. (**c**) The stimulus (normalized to zero mean and unit variance) is shown for reference as a function of time.

The brain does not have direct access to the stimulus, but only receives codewords 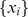, drawn from 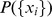, by the retina. It is therefore useful to estimate for every moment in time, the *surprise* about the output of the retina, and thus about the stimulus, which is given by 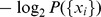. We, as experimenters—but not the brain—have access to stimulus repeats and thus to 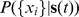, so we can compute the average value of surprise (per unit time) at every instant 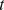 in the stimulus:
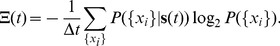
(8)This quantity can be expressed using the entropies and the learned parameters of our maximum entropy models, and is plotted as a function of time in Fig. 10. Since averaging across time is equal to averaging over the stimulus ensemble, we see from Eq. (8) that 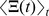 would have to be identically equal to 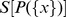 under the condition that 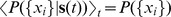 (marginalization). Since we build models for 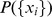 (static ME) and 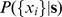 (S2) from data independently, they need not obey the marginalization condition exactly, but they will do so if they provide a good account of the data. Indeed, by using the static ME and S2 distributions in Eq. (8) for surprise, we find that 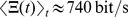, very close to the entropy rate 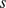 of the total vocabulary and within the estimated error bars of the entropy, which are 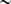
1%.

To estimate the information transmission, we have to subtract the noise entropy rate from the output entropy rate 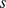, as dictated by Eq. (7). The entropy of the S2 model is an upper bound on the noise entropy; since this is not a lower bound, we cannot put a strict bound on the information transmission, but can nevertheless estimate it. Fig. 10 shows the “instantaneous information” [64], 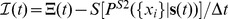, as a function of time; from Eq. (7), the mutual information rate is a time average of this quantity, 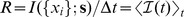. We find 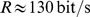. This quantity can be compared to the total entropy rate of the stimulus itself (which must be higher than 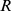), which in our case is 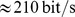 (see Methods). While our estimates seem to indicate that a lot of vocabulary bandwidth (730 bit/s) is “lost” to noise (600 bit/s), the last comparison shows that the Gaussian FFF stimulus source itself is not very rich, so that the estimated information transmission takes up more than half of the actual entropy rate of the source.

Lastly, we asked how important is the inclusion of pairwise interactions, 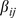, into the S2 model, compared to the S1 model, when accounting for information transmission. We therefore estimated the noise entropy rate for the S1 model, 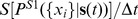, which was found to be 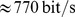, considerably higher than the noise entropy of the S2 model. Crucially, this noise entropy rate is larger than the total entropy rate 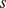 estimated above, which is impossible for consistent models of the neural codebook and the vocabulary (since it would lead to negative information rates). This failure is a quantitative demonstration of the inability of the uncoupled LN models to reproduce the statistics of the population vocabulary, as shown in Fig. 6b, despite a seemingly small performance difference on the level of single cell PSTH prediction.

## Discussion

We presented a modeling framework for stimulus encoding by large populations of neurons, which combines an individual neuronal receptive field model, with the ability to include pairwise interactions between neurons. The result is a stimulus-dependent pairwise maximum entropy (S2) model, which is the most parsimonious model of the population response to the stimulus that reproduces the linear-nonlinear (LN) aspect of single cells, as well as the pairwise correlation structure between neurons. In two limiting cases, the S2 model reduces to known models: if the single cell parameters 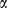 are static, S2 becomes the static pairwise maximum entropy model of the population vocabulary; if the couplings 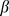 are 0, S2 reduces to S1, the set of uncoupled LN models.

We applied this modeling framework to the salamander retina presented with Gaussian white noise stimuli, and found that the interactions between neurons play an important role in determining the detailed patterns of population response. In particular, the S2 model gave better prediction of PSTH of single cells, yielded orders-of-magnitude improvement in describing the population patterns, and captured significant aspects of noise correlations. The deviations between the S2 and the S1 model became significant for 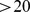 cells, and tended to occur at “interesting” times in the stimulus, precisely when the neural population was not silent.

The S2 model allowed us to improve over LN models for salamander retinal ganglion cells in terms of the PSTH prediction of single cells. But, more importantly, it gave a huge improvement in terms of describing and predicting the population activity patterns, or codewords. Interestingly, for parasol cells in the macaque retina under flickering checkerboard stimulation, the generalized linear model did not yield firing rate improvement relative to uncoupled LN models (but did improve the prediction of higher order statistics of neural activity) [41]. In both cases, however, the improvements reflect the role of dependencies among cells in encoding the stimulus, and their effect becomes apparent when we ask questions about information transmission by a neural population. Maximum entropy models can only put upper bounds on the total entropy and the noise entropy of the neural code (and this statement remains true even if successive codewords are not independent), and as such cannot set a strict bound, but only give an estimate, for the information transmission. Nevertheless, ignoring the inter-neuron dependencies by using the S1 model would predict the total population responses so badly that the estimated noise entropy would be higher than the upper bound on the total entropy, which is a clear impossibility. In contrast, S2 model gives noise entropy rates that are consistent with the estimate from the static maximum entropy model, and transmission rates that amount to about 60% of the source entropy rate (comparable to estimates of coding efficiency in single neurons, e.g., Ref. [65]).

An alternative approach to describing the joint response of large populations of neurons to external stimuli has been presented in Ref [41]. The Generalized Linear Model (GLM) gives a generative model from which one can sample simulated responses to new stimuli, relying on activity history and temporal dependencies between cells. The crucial assumption of the GLM is that the responses of the neurons are conditionally independent given the stimulus and the spiking history; to satisfy this assumption, the discretization of time has to be as fine grained as possible, but certainly well below the discretization of 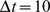 or 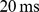 typically used for maximum entropy models in our retinal preparation. This conditional independence, guaranteed by very short time bins, allows tractable inference procedures to be devised for fitting the GLMs from data. On the other hand, it makes—by its very definition—successive activity patterns dependent on each other, because that is the only way to introduce interactions between the spikes. In contrast, maximum entropy models pick the time bin to be short enough such that multiple spikes are rarely observed in the same time bin, but long enough so that most of the strong spike-spike interactions (as well as fine temporal detail, such as spike-timing jitter) occur *within a single bin*. This allows us to view activity patterns in successive time bins as codewords (although some statistical dependence between them remains: in the SDME models this is probably due to multiple timescales on which the neurons respond to stimuli; and in the static ME model [31] due to, in part, stimulus correlation). If we were to make the time scale in maximum entropy models much shorter, e.g. by an order of magnitude or more, we could make the conditional independence assumption of the responses given the stimuli *and* previous spiking. This would lead us to GLM-like models in the maximum entropy framework, e.g., to dynamic/nonequilibrium generalizations of Ising models [48]; in this case, however, we would again lose the interpretation where the instantaneous state of the retina is represented well by a single codeword. For this reason, GLM and SDME are complementary approaches: the first allows for a temporally-detailed probabilistic description of a spiking process, while the second gives an explicit expression for the probability distribution over codewords in longer temporal bins. To our knowledge, there is no easy way to derive one model from the other: while one can fit the GLM with a very small time bins, use it to *generate* rasters and re-discretize those into time bins of longer duration to get a codeword representation, building a probabilistic model for the codewords from the GLM-derived rasters is as difficult as building it for original data. While a more detailed comparison of these models is beyond the scope of the current work, it is interesting to note that these approaches are different and complementary also in terms of the potential interpretation of their parameters: GLM couplings between neurons have an intuitive interpretation in terms of causal dependency between cells, whereas the SDME ones suggest a prior on the coding vocabulary of the population (see below). Finally, from a modeling viewpoint, GLM lends itself to a clean and tractable maximum likelihood inference framework with regularization, whereas the SDME offers the tools and insights of statistical physics [27], [42]–[53] (including, e.g., advanced Monte Carlo schemes for entropy estimation [66] and the partitioning of the space of codewords in terms of metastable states briefly discussed in this paper).

Tkačik and colleagues [62] have suggested that one can interpret 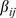 in an SDME model as a prior over the activity patterns that the population would use to optimally encode the stimulus. For low noise level they argued that the prior should be “weak” (and could help decorrelate the responses) because the population could faithfully encode the stimulus, whereas in the noisy regime, the prior should match the statistics of the sensory world and thus counteract the effects of noise. Berkes and colleagues [67] suggested a similar reason for the relationship between ongoing and induced activity patterns in the visual cortex. Our results show that interactions are necessary for capturing the network encoding, and implicitly reflect the existence of such a prior. The recovered interactions are strongly correlated with the interaction parameters of a static, stimulus independent model over the distribution of patterns, making it possible for the brain (which only has access to the spikes, not the stimulus) to learn these values. Whether the interactions are matched to the statistics of the visual inputs as suggested in Ref [62] will be the focus of future work.

The maximum entropy models presented here can be immediately applied to other brain areas where one can get stable recordings of many neurons over a few tens of minutes [35], [68]. SDME could be applied to spatially structured stimuli, for instance, to capture the response to the flickering checkerboards: obtaining good estimates of the spatio-temporal receptive fields is standard procedure, identical to that in LN or GLM-type models, while fitting the parameters 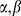 of the SDME is equally tractable on full field flicker (as presented here) or movie with spatial structure. In practice, a different tradeoff would be chosen in experimental design, by making stimulus segment longer to sample the linear filters better from many different stimuli, and decreasing the number of repeats. As we noted above, for fitting the model, one could also eliminate repeated structure altogether, yet repeated presentations of the same stimuli would still be needed to assess the model quality in terms of the PSTH. The current design of the experiment focused on a very large number of repeats of the same stimulus, to allow for as accurate estimate of the PSTH and correlations of individual cells, while future experiments could allow for evaluation of the model on novel repeated stimuli. Given the results we have presented here and those of [41], we expect that the SDME models would significantly outperform the LN models on novel stimuli as well. Other potential extensions of the pairwise SDME model would include temporal dependencies as in Refs [31], [49] or a SDME model where the pairwise interactions are also stimulus dependent. While it is not immediately clear how such dependency would be expressed for the 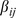 (unlike the linear filter description of the single cell parameters, 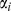
's), such a model would be instrumental for analysis of population adaptation or learning. Another extension would be to include the dependence of 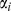 on multiple stimulus projections, or to include high-order interaction terms between spikes, which are likely to play an important role for large populations responding to natural stimuli [34], [35]. Finally, we also expect that sampling from larger populations, as future experiments will allow, would enable us to give a full characterization of the interaction maps between cells of different classes, which would most likely reflect independence between classes with strong correlations between the cells of the same class, or even stronger correlations at the population level including across different classes; the two alternatives represent an exciting (and still mostly unanswered) question. We expect that increasingly detailed statistical models of neural codes, and the efforts to infer such models from experimental data, will allow us to focus our attention on population-level statistics and on finding principled information-theoretic measures for quantifying the code, like the surprise and instantaneous information suggested here.

## Methods

### Electrophysiology

Experiments were performed on the adult tiger salamander, *Ambystoma tigrinum*. All experiments were in accordance with Ben-Gurion University of the Negev and government regulations. Extracted retinas were placed with the ganglion cell layer facing a multielectrode array with 252 electrodes (Ayanda Biosystems, Switzerland), and superfused with oxygenated Ringer medium at room temperature. Extracellularly recorded signals were amplified (MultiChannel Systems, Germany) and digitized at 10 kHz, and spike-sorted using custom software written in MATLAB.

### Visual stimulation

Stimuli were projected onto the retina from a CRT video monitor (ViewSonic G90fB) at a frame rate of 60 Hz; each movie frame was presented twice, using standard optics. Full Field Flicker (FFF) stimuli were generated by independently sampling spatially uniform gray levels (with a resolution of 8 bits) from a Gaussian distribution, with mean luminance of 147 lux and the standard deviation of 33 lux. These data allow us to estimate the entropy rate of the source (as used in the main text), by multiplying the entropy of the luminance distribution with the refresh rate. To estimate the cells' receptive fields, checkerboard stimulus was generated by selecting each checker (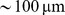 on the retina) randomly every 33 ms to be either black or white. To identify the RF centers, a two-dimensional Gaussian was fitted to the spatial profile of the response. The movies were gamma corrected for the computer monitor. In all cases the visual stimulus entirely covered the retinal patch that was used for the experiment.

### Estimating model statistics from data

The firing rates of the cells and the overall covariance of the spiking activity are the key statistics for inferring the models we present, so the reliability of our estimates for these quantities is a key systematic issue. Previous work has shown that 10–20 minute recordings give very reliable estimates [35], [68], and that train data of similar size allows for reliable estimates of pairwise-maximum-entropy-based models for populations of this size [68]. The error on instantaneous firing rate was estimated by splitting 626 repeats into two random halves 50 times, and estimating firing rate for each neuron. The relative error in the firing rate, 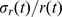, estimated as (relative) std over random splits of data, scales tightly with the mean firing rate with the power 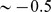, such that at instantaneous rates of about 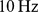 the error is 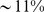, at 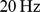 the error is 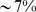, and at 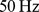 the error is 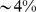. For correlations, we assess their significance by comparing the distribution of real correlation coefficients to the (null) distribution where the spikes for each neuron have been randomized in time. The null distribution is evaluated over one half of the repeats, because this is the data size used for training; the mean of the distribution is 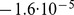, and the std 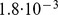, making 95% of observed correlations larger than this spread due to sampling. More in detail, the relative error on correlations was assessed by splitting data 50 times randomly into two halves, and seeing that the relative error scales with the value of the correlations 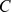, so that the typical error at significance threshold was about 60%, for 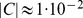 (80% of all correlations) it was 18%, for 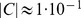 (23% of all correlations) it was 4%, and for 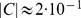 it was less than 2%. The average error on significant correlations is slightly below 10%. To quantify the stability of the recordings across time, we computed for each cell the approximate drift in the firing rate, by linearly regressing the average firing rate in each repeat against the repeat index. For about half of the cells the relative change in the firing rate across the whole duration of the experiment was below 25% (average 14%), while for 80% of the cells the drift was below 50% (average 24%). To deal with the remaining non-stationarity, we selected as our training data all odd numbered repeats, and for our test data all even numbered repeats, so that the models were trained and tested across the non-stationary behavior.

### Inferring SDME from data

The LN model for each neuron 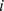 consists of the linear filter 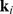, and the nonlinear function 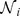, which is defined pointwise on a set of binned values for the generator signal, 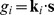. We used binning into 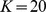 bins such that initially each bin contains roughly the same number of values for 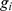, but subsequently the binning is adaptively adjusted (separately for each neuron) to be denser at higher values of 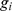, where the firing rates are higher. We fitted LN models with varying number of 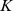 bins, and have chosen 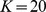 when the performance of the LN models appeared to saturate [69].

To find the parameters of the stimulus-dependent maximum entropy model (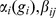), we retained the binning of the generator signal used for LN model construction. Given trial values for the SDME parameters, we estimated the chosen expectation values (covariance matrix 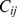 of neural activity, and the firing rate conditional on 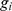, 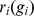) by Monte Carlo sampling from the trial distribution in Eq. (4); the learning step of the algorithm is computed by comparing the expectation values in the trial distribution and the empirical distribution (computed over the training half of the stimulus repeats). In detail, we used a gradient ascent algorithm, applying a combination of Gibbs sampling and importance sampling in order to efficiently estimate the gradient, by using optimizations similar to those described in Ref. [70]. Sampling was carried out in parallel on a 16 node cluster with two 2.66 GHz Intel Quad-Core Xeon processors and 16 GB of memory per node. The calculation was terminated when the average error in firing rates and coincident firing rates reached below 1% and 5% respectively, which is within the experimental error.

To compute the single neuron PSTH and compare the distributions of codewords from the model to the empirical distribution, we used Metropolis Monte Carlo sampling to draw codewords from the model distributions; we drew 5000 independent samples (to draw uncorrelated configurations, a sample was recorded only after 100 “spin-flip” trials) for every timepoint, for a total of 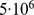 samples; the same procedure was used also to draw from the conditionally independent (T1,S1) models. To estimate the entropies of high dimensional SDME distributions, we used the “heat capacity integration” method, detailed in Ref [32]. Briefly, a maximum entropy model 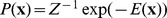 (where 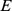 is the Hamiltonian function determined by the choice of constrained operators and the conjugated parameters) is extended by introducing a new parameter 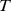, much like the temperature in physics, so that 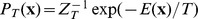. The entropy of the distribution is given by 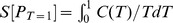, where the heat capacity 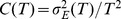, and the variance in energy can be estimated at each 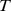 by Monte Carlo sampling. In practice, we run a separate Monte Carlo sampling for a finely discretized interval of temperatures, 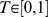, estimate 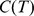 for each temperature, and numerically integrate to get the entropy 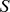. We have previously shown that this procedure yields robust entropy estimates even for large numbers of neurons [27], [32].

### Evaluating the likelihood and goodness of fit

To evaluate the performance of the models on the testing data, we computed (i) the average per-codeword log-likelihood (reported in the Results section), and (ii) the GoF (goodness-of-fit) figure, reported in Fig. 6. Regarding (i), for model 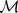 the log-likelihood is 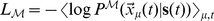, where the average is over all testing repeats 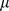 and all times 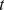. For models S1, S2, the evaluation is straightforward. For T1 model, there is a problem whenever the firing rate of a neuron in the training set is 0, which leads to undefined log likelihoods. To address this, we add a small regularizer 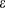 to the estimated firing rates that define the T1 model, and choose 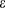 to maximize the log-likelihood of T1 on the test set, thus giving maximal possible advantage to the T1. We also created two models by empirically sampling the frequencies of codewords on training (testing) data. Sampling the frequencies on testing data and evaluating on testing data gives the absolute lower bound to the log likelihood. When the frequencies are sampled on training data, we again face a possible problem for codewords whose empirical probability is 0, but which occur in test data. We introduce a pseudocount regularizer to give these codewords non-zero probability, and set the regularizer to maximize the log-likelihood on testing data, again maximally favoring this model. Regarding (ii), we compute GoF (goodness-of-fit) figure as std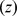, where 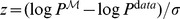. 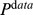 is the empirical probability of a codeword on the test set, 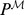 is its model probability, 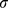 is the expected error on 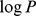, computed from the multinomial variance for every codeword given its empirical probability, and the std is taken over all non-silent patterns of the test set plotted in Fig. 6, top row.
